# Effect of Different Head-Neck Postures on the Respiratory Function in Healthy Males

**DOI:** 10.1155/2018/4518269

**Published:** 2018-07-12

**Authors:** Hamayun Zafar, Ali Albarrati, Ahmad H. Alghadir, Zaheen A. Iqbal

**Affiliations:** ^1^Rehabilitation Research Chair, College of Applied Medical Sciences, King Saud University, Riyadh, Saudi Arabia; ^2^Department of Rehabilitation Sciences, College of Applied Medical Sciences, King Saud University, Riyadh, Saudi Arabia

## Abstract

Normal respiration is a very intricate function that comprises mechanical as well as nonmechanical components. It is shown to be affected by various factors including age, lifestyle, disease, and change in posture. With the increased use of hand held devices, everyone is prone to poor sitting postures like forward head posture. The purpose of this study was to evaluate the effect of assumed forward head posture and torticollis on the diaphragm muscle strength. A sample of 15 healthy males, aged 18-35 years, was recruited for this study. All subjects performed spirometry to measure the forced expiratory volume in 1 second (FEV_1_), the forced vital capacity (FVC), and FEV_1_/FVC ratio. SNIP was measured during upright sitting, induced forward head posture, and torticollis. Subject's mean age (SD) was 23(6) years. The SNIP score of the subjects during sitting with FHP was lower as compared to that during upright sitting. It decreased significantly during induced right torticollis position. This is the first study exploring the impact of different head and neck positions on respiratory function. Alteration of head and neck positions had an immediate negative impact on respiratory function. Clinicians should be prompted to assess respiratory function when assessing individuals with mal-posture.

## 1. Introduction

Normal respiration is a very intricate function comprising mechanical as well as nonmechanical components. It can be affected by various factors, including age, lifestyle, disease, and change in posture, that can interfere with its normal functioning [1, 2]. Different postures such as forward head posture (FHP) and kyphosis have been shown to alter breathing mechanism including diaphragm mobility [3–7]. Alteration of cervicothoracic mobility impairs normal breathing mechanics by reducing diaphragm mobility and strength [8].

With the introduction of modern technologies including new age computers and other communication devices, such as hand held devices that are available to everyone even youths, individuals are frequently using these devices in bad sitting postures. These include FHP and rotated neck posture similar to torticollis position which in turn affects their breathing pattern and consequently may lead to breathing dysfunction. Similar postures are usually seen in elderly as a result of normal aging process and related to neck disorders [9]. Furthermore, musculoskeletal problems of the shoulder, neck, and back can cause twisting of the neck to one side known as torticollis [10, 11]. These positions can interfere with the breathing mechanism and alter the diaphragm mobility [7, 12].

Although there are various studies that have examined the effect of sitting, lying, and standing postures on respiratory function, there has been no study that examines the effect of altered head and neck posture on respiratory muscles strength in healthy young subjects with no prior deformity or disease. It would be interesting to know how altered head-neck postures would affect various breathing parameters. This research aimed to evaluate the effect of assumed FHP and torticollis on the diaphragm muscle strength.

## 2. Materials and Methods

A sample of 15 healthy males, aged 18-35 years, was recruited for this study. They were screened for any cardiopulmonary disease, sinusitis, or any type of pain or postural abnormality and were excluded if any positive sign or symptom was found. All the subjects were informed about the purpose and nature of this study and their informed consent was obtained before participation. Ethical approval in compliance with the Helsinki Declaration was obtained from Rehabilitation Research Review Board, King Saud University (Ref. no. KSU/RRC/72).

### 2.1. Body Composition

Height (cm) and weight (kg) were measured using stadiometer while standing barefoot and wearing light weighted clothes, and body mass index was calculated (kg/m^2^).

### 2.2. Lung Function Measurement

All subjects performed spirometry (Vitalograph Alpha, Bucks, UK) to measure the forced expiratory volume in 1 second (FEV_1_), the forced vital capacity (FVC), and FEV_1_/FVC ratio.

### 2.3. Sniff Nasal Inspiratory Pressure (SNIP)

During upright sitting, SNIP was measured using MicroRPM device (MicroRPM, Micro Medical, Ltd., Kent, UK) [13]. Subjects seated normally with back fully supported and arms resting on thighs, a proper probe was inserted in one of the nostrils, and subjects were asked to take a sharp and quick sniff from functional residual capacity (FRC). This was repeated at least five times or until subject could not score more than 10 cm H_2_O, and the maximum value was chosen for analyses [14].

### 2.4. Measurement during Induced Forward Head Posture

The subjects were asked to assume the FHP by bending their upper back and protruding their head such that tragus of ear lies ahead of shoulder level [11, 15]. They were instructed to move their head forward and maintain horizontal gaze to avoid head bending [16–18]. While subjects maintained this position, a proper probe was inserted in a nostril and subject made a sharp and quick sniff from FRC. The procedure was repeated at least five times or until subject could not score more than 10 cm H_2_O, and the maximum value was chosen for analyses.

### 2.5. Measurement during Induced Torticollis

The subjects were asked to rotate their head to the left side and side flex to the right side. In this position, the SNIP test was repeated as mentioned above and highest value was chosen for the analysis.

### 2.6. Statistical Analyses

Data were analyzed using GraphPad InStat 3.0 (GraphPad Software Inc., CA, USA). Data were examined for normality prior to analysis. Mean and standard deviations were used to summarize the data. Repeated Measures Analysis of Variance with post hoc (Bonferroni) test was used to examine the differences between SNIP scores during upright sitting, sitting with FHP, and sitting with induced torticollis. The values were considered significant at p values less than 0.05.

## 3. Results

Subject's mean age (standard deviation) was 23(6) years while BMI was 26(5) kg/m^2^ (Table 1). The SNIP score of the subjects during upright sitting was 93(26) cmH_2_O (Table 2). There was no association between the SNIP scores, age, or BMI.

The SNIP score of the subjects during sitting with FHP was lower compared to that during upright sitting (p<0.001). The SNIP score decreased significantly during induced right torticollis compared to upright sitting posture (p<0.001). There was no significant difference between the SNIP score in FHP and torticollis position (p>0.05) (Table 2).

## 4. Discussion

This is the first study exploring the impact of different head and neck positions on respiratory function. Our results showed that alteration of head and neck postures had an effect on respiratory function. The assumed FHP and torticollis postures decreased the SNIP values compared to the normal upright sitting.

In present study, we found that alteration of the head-neck position had an immediate effect on respiratory function characterized by reduced diaphragm strength. This indicates the huge impact the head position can have on physio-mechanical function of the respiratory system. Sustaining poor ergonomic postures while using computers, TV, video games, mobile technologies, etc. for a prolonged period of time can lead to development of FHP [19]. It is often unnoticed at early stages until symptoms appear. Continuous repetitive adaptation of these positions turns into a deformity causing neck and upper back pain, stiffness, shallow breathing, and breathing dysfunction. Individuals with FHP showed reduced diaphragmatic strength as a result of its reduced activity [20]. This is supported by work of Hodges and colleagues who reported that the diaphragm mobility is altered by posture movements [7]. Increased degree of FHP has a bearing effect on chest expansion and respiratory muscles activities which can lead to reduced alveolar ventilation [12]. A number of studies have found that patients with neck pain had lower respiratory capacity and function [21]. Unlike patients with neck disorder, our results showed that even in healthy subjects, with no previous history of mechanical or physiological dysfunction, induced FHP resulted in an immediate effect on respiratory function even when subjects assume FHP for a short duration of time. This could be a result of temporary entrapment of the phrenic nerve, which supplies the diaphragm, reduces it neural activity, and consequently weakens the diaphragm activity [22]. Not limited to this, altered diaphragm function leads to core muscles instability, which will further lead to other systemic and musculoskeletal disorders including spinal instability [23, 24].

Approximation of ribs and pelvis in subjects with slumped and kyphotic posture has been shown to increase intra-abdominal pressure making movement of diaphragm difficult, leading to reduced lung capacity and inspiratory flow [2]. Similarly in patients with FHP and torticollis, flattening of normal cervical curve and development of secondary round upper back [25] compress the chest cavity which can alter breathing capacity.

During torticollis, there is shortening of sternocleidomastoid, trapezius, and other neck muscles [26, 27]. This shortening is more on one side causing side flexion and rotation of head [28]. These muscles are also accessory muscles of respiration, which are used in addition to the diaphragm [29]. These help to elevate rib cage especially during labored breathing [30, 31]. Weakness of sternocleidomastoid, scalene, and trapezius has been associated with decrease in maximal inspiratory and expiratory pressure [32]. Neck muscle weakness associated with neck pain has been shown to decrease thoracic mobility affecting pulmonary function [33]. Patients with acute torticollis are usually unable to take a proper breathing and therefore this issue should be addressed when examining patients with acute torticollis.

### 4.1. Limitations

We saw the effect of head and neck posture, induced for a short period of time, on respiratory function in healthy male subjects, so we cannot extrapolate these results on healthy females. This study could be repeated in patients with such deformities acquired over a large period of time and determine if management could reverse negative impacts on respiratory system.

## 5. Conclusions

Alteration of head and neck positions can have an immediate negative impact on respiratory function. Clinicians should be prompted to assess respiratory function when assessing individuals with FHP and torticollis and reduce the tension on respiratory system to avoid consequences. The SNIP is a simple tool and easy to use and should be integrated into practice for screening individuals with mal-posture.

## Figures and Tables

**Table 1:** Demographic characteristics of participants (n=15).

	**Mean (SD)**

**Age (years)**	23 (6)

**Height (cm)**	170 (5)

**Weight (Kg)**	74.6 (16)

**BMI (Kg/m** ^**2**^ **)**	25.6 (5.04)

**FEV1**	0.92 (0.13)

FVC	0.85 (0.09)

**FEV1/FVC ratio**	1.15(0.17)

**Table 2:** SNIP: Sniff nasal inspiratory pressure (cmH_2_O) in different head and neck positions (n=15).

**Position**	**Mean (SD)**	**Significance**
**SNIP (upright sitting)**	93.46 (25.82)	p<0.001
**SNIP (FHP)**	80.80 (24.42)	

**SNIP (upright sitting)**	93.46 (25.82)	p<0.001
**SNIP (torticollis)**	80.93 (22.20)	

**SNIP (FHP)**	80.80 (24.42)	p>0.05
**SNIP (torticollis)**	80.93 (22.20)	

## Data Availability

The data used to support the findings of this study are available from the corresponding author upon request.

## Abbreviations

FHP: Forward head posture
FEV_1_: Forced expiratory volume in 1 second
FVC: Forced vital capacity
SNIP: Sniff nasal inspiratory pressure
FRC: Functional residual capacity.
